# Association of Regional Variation in Primary Care Physicians’ Colorectal Cancer Screening Recommendations with Individual Use of Colorectal Cancer Screening

**Published:** 2007-09-15

**Authors:** Jennifer S Haas, Garrett Fitzmaurice, Phyllis Brawarsky, Su-Ying Liang, Robert A Hiatt, Kathryn A Phillips, Carrie N Klabunde, Martin L Brown

**Affiliations:** Division of General Medicine and Primary Care, Brigham and Women’s Hospital and Harvard Medical School, Boston, Massachusetts; Brigham and Women’s Hospital, Boston, Massachusetts; Brigham and Women’s Hospital, Boston, Massachusetts; University of California, San Francisco, California; University of California, San Francisco, California; University of California, San Francisco, California; National Cancer Institute, Bethesda, Maryland; National Cancer Institute, Bethesda, Maryland

## Abstract

**Introduction:**

Studies show that the recommendations of a primary care physician for colorectal cancer screening may be one important influence on an individual's use of screening. However, another possible influence, the effect of regional differences in physicians' beliefs and recommendations on screening use, has not been assessed.

**Methods:**

We linked data from the National Health Interview Survey on the use of colorectal cancer screening by respondents aged 50 years or older, by hospital-referral region, with data from the Survey of Colorectal Cancer Screening Practices on the colorectal cancer screening recommendations of primary care physicians, by region. Our principal independent variables were the proportion of physicians in a region who recommended screening at age 50 and continuing screening at the recommended frequency.

**Results:**

On average, 53.3% of physicians in a region correctly recommended initiating colorectal cancer screening, and 64.8% advised screening at the recommended frequency. Of adults who lived in regions where less than 30% of physicians correctly recommended initiating screening, 47.3% had been screened, in contrast to 54.8% in areas where 70% or more of physicians made correct recommendations. Seventy-one percent of respondents living in regions where less than 30% of physicians advised screening at the recommended frequency were current on screening, in contrast to 79.9% of respondents living in regions where 70% or more of physicians made this recommendation. These differences were statistically significant after adjustment for individual characteristics.

**Conclusion:**

Strategies to improve colorectal cancer screening recommendations of primary care physicians may improve the use of screening for millions of Americans.

## Introduction

Mortality from colorectal cancer (CRC), the third most common cancer in the United States, can be prevented by early detection (1). For this reason, screening for CRC is strongly endorsed by national professional societies and expert panels (2-5). Despite the public health importance of CRC screening, however, it remains widely underused (6,7). Limited patient awareness and lack of physician recommendations during a health care visit are both barriers to CRC screening (8-11). Because several established options for CRC screening exist (e.g., fecal occult blood testing [FOBT], sigmoidoscopy, colonoscopy), physicians may be unsure about how best to implement screening. Survey data show that primary care physicians commonly report CRC screening practices that are inconsistent with current guidelines (12). Screening practices may, therefore, vary by region (13).

The goal of our analysis was to examine whether regional variations in the beliefs and recommendations of primary care physicians about CRC screening are associated with regional levels of screening use. The conceptual framework for this study is derived from the expanded behavioral model of health care that incorporates the role of contextual variables on health care use (14,15). We hypothesized that people living in an area where more primary care physicians recommend CRC screening consistent with national guidelines would be more likely to use and be current on screening, after accounting for the individual characteristics associated with screening use.

## Methods

### Data 

Our analysis is based on data from the 2000 and 2003 National Health Interview Surveys (NHIS) and the National Cancer Institute's (NCI) 1999–2000 Survey of Colorectal Cancer Screening Practices (SCCSP), Primary Care Physician Questionnaire (12). The NHIS, conducted by the National Center for Health Statistics of the Centers for Disease Control and Prevention (CDC), is nationally representative and collects information about demographic characteristics, chronic health conditions, health insurance, and health behaviors of the civilian, noninstitutionalized U.S. population (www.cdc.gov/nchs/nhis.htm). The NHIS Cancer Control Supplement, administered in 2000 and 2003, includes a series of questions about the use of CRC screening (16).

The SCCSP, conducted by NCI, CDC, and the Centers for Medicare and Medicaid Services, surveyed a nationally representative sample of 1235 practicing primary care physicians for 1999–2000, including family and general practitioners, general internists, and obstetricians and gynecologists (8,12). The survey was designed to estimate CRC screening capacity and the knowledge and beliefs of primary care physicians about CRC screening. Details of the sampling scheme and a description of the characteristics of the respondents have been published (12).

We merged data from the NHIS with data from the SCCSP at the county level. These data were then aggregated to hospital-referral regions, which represent regional health care markets for medical care and have been used extensively to examine regional variation in health care use (17-24). The United States has 306 hospital-referral regions (17). We constructed independent variables to reflect the recommendations of primary care physicians in the region where each respondent lived. Because of the confidential nature of these data, analyses were conducted at the Research Data Center of the National Center for Health Statistics.

### Sample 

We included data on individuals from the NHIS who were aged at least 50 years, had not previously received a diagnosis of CRC, and responded to questions regarding the use of CRC screening. Because the sampling frames of the NHIS and the SCCSP were not identical, we limited our sample to individuals who lived in a hospital-referral region where four or more primary care physicians were surveyed in the SCCSP (N = 12,727 individuals in 122 hospital-referral regions).

### Outcome variables 

NHIS respondents were asked several questions about their use of CRC screening: if they had ever had an FOBT using a home test kit and, if so, the timing of their most recent home FOBT; if they had ever had a CRC screening test by sigmoidoscopy or colonoscopy and, if so, the type of test and the timing of their most recent test. Individuals were classified as "ever screened" for colorectal cancer if they reported ever taking a home FOBT, having had a sigmoidoscopy, or having had a colonoscopy. We also examined whether subjects who had reported CRC screening were current on screening, (i.e., home FOBT during the past year, sigmoidoscopy within the past 5 years, or colonoscopy within the past 10 years [2]). NHIS did not ask about barium enema. Although the American Cancer Society includes double contrast barium enema every 5 years as an acceptable screening option (3), the U.S. Preventive Services Task Force did not find direct evidence that this method is effective in reducing CRC mortality (2).

### Independent variables 

We used data from the NHIS to define individual characteristics and included age, sex, race and ethnicity, education, health insurance, health-care-seeking behavior, prior history of cancer other than CRC, number of chronic medical conditions, and number of behavioral risk factors for CRC. We categorized ethnicity as non-Hispanic white, non-Hispanic black, Hispanic, or other race and ethnicity. Educational attainment was defined as less than high school graduation, high school graduate, some college, and college graduate. Health insurance categories were uninsured; Medicare with private supplemental insurance, or private insurance; Medicare without supplemental coverage; and Medicaid or dual eligibility for Medicare and Medicaid. We categorized health-care–seeking behavior according to whether an individual had a usual source of health care, evidenced by a visit to any health care professional, including a dentist, in the past year. Chronic medical conditions included arthritis, peptic ulcer disease, chronic lung disease, cardiovascular disease, hypertension, and diabetes. Behavioral risk factors for CRC included current cigarette use, heavy drinking (consuming 60 or more alcoholic drinks per month for men and 30 or more for women), and lack of regular exercise (25-27).

Primary care physicians who participated in the SCCSP were asked at what age and how frequently they recommended each CRC screening method for a patient at average risk (12). Physicians reporting the use of FOBT were asked whether they provided office-based or home tests. Because sensitivity is lower for a single office-based FOBT than for the home test, in which samples are collected over 3 days (2,4), only a home test was considered adequate screening. We coded recommendations for initiation of each type of CRC screening as being in accordance with screening guidelines if the physician reported recommending that patients begin having the test at age 50 (2). We coded each physician's belief about frequency of screening as being in accordance with the guidelines if the response was at the recommended interval for at least one type of CRC screening test. We aggregated all responses according to hospital-referral region to create two region-level measures of CRC screening practices: 1) the proportion of primary care physicians in a hospital-referral region who recommended initiating CRC screening at age 50 years, and 2) the proportion of these physicians who advised at least one screening test at the recommended interval. These two variables captured distinct information supported by a correlation coefficient of only −0.04.

### Statistical analysis 

Using the data on the individual as the unit of analysis, we constructed multilevel logistic regression models to examine the odds of undergoing CRC screening. We based the models on the average proportion of primary care physicians in an individual's hospital-referral region who recommended CRC screening, after controlling for individual factors associated with CRC screening. To reflect the greater precision of estimates from hospital-referral regions with a large number of primary care physicians responding, we adjusted NHIS survey sample weights for the number of primary care physicians per region. The odds ratios (OR) for region-level measures of physician recommendations were expressed for a 30-percentage-point increase in the proportion of primary care physicians in the region recommending CRC according to the guidelines. Models accounted for the clustering of individuals in regions and for the survey sample weights and were estimated with SAS 9.0 (SAS Institute, Cary, North Carolina). We based independent variables on prior work and on their statistical relationships with the dependent variable. We hypothesized that regional physician belief about the age at initiation of CRC screening would be associated with the likelihood that an individual living in a region would ever be screened. We also hypothesized that regional physician recommendations about screening intervals would be associated with the likelihood that a patient would be current on screening and included this variable in this model. The final models included age, sex, race and ethnicity, education, insurance, usual source of care, prior diagnosis of cancer other than CRC, dental visit within the prior year, number of chronic health conditions, number of behavioral risk factors for CRC, year of NHIS survey, and relevant hospital-referral region measure.

## Results

### Factors associated with ever receiving CRC screening 

Only 50.2% of adults aged 50 years or older had ever been screened for CRC (Table 1). Hispanics were significantly less likely than non-Hispanic whites to have been screened. Respondents with less than a college degree were less likely than college graduates to have been screened. Uninsured respondents, those who had Medicare without supplemental coverage, and those with Medicaid or who were dually eligible for Medicare and Medicaid were less likely than those with private insurance or Medicare plus a supplemental policy to have ever been screened. Respondents without a usual source of care were less likely than those with one to be screened. Respondents who had previously received a diagnosis of cancer were more likely than those with no diagnosis to have been screened. CRC screening increased with the number of chronic conditions, but decreased as the number of behavioral risk factors for CRC increased. CRC screening increased between 2000 and 2003.

On average, 53.3% of primary care physicians in a hospital-referral region recommended initiating CRC screening at age 50 (range 0%–100%). In regions where less than 30% of physicians recommended initiating screening at age 50, 47.3% of respondents had been screened, in contrast to 54.8% of respondents in regions where 70% or more of physicians made this recommendation. After adjustment for individual characteristics, an absolute increase of 30 percentage points (e.g., from 50% to 80% or 20% to 50%) in the proportion of primary care physicians in a hospital-referral region who recommended initiating CRC screening at age 50 was associated with a higher prevalence of screening in that region (OR, 1.09; 95% CI, 1.01-1.18).

### Factors associated with current CRC screening 

Among respondents who had ever received CRC screening, 77.9% were current on screening (Table 2). Women were less likely than men to be current on screening. Respondents who had some college education were less likely than those who had graduated from college to be current on screening. We found no association between current CRC screening and race and ethnicity, insurance, prior diagnosis of cancer other than CRC, the number of chronic health conditions, or the number of behavioral risk factors for CRC. Respondents without a usual source of care were less likely than those with one to be current on screening. The proportion of respondents who had been screened and were current on screening increased between 2000 and 2003.

On average, 64.8% of primary care physicians in hospital-referral regions recommended at least one CRC screening test at the recommended interval (range 0%–100%). In regions where <30% of physicians advised screening at the recommended frequency*,* 70.7% of respondents were current on screening, in contrast to 79.9% of respondents living in areas where ≥70% of physicians made this recommendation. After adjustment, an increase of 30 percentage points in the proportion of primary care physicians in a hospital-referral region who recommended at least one CRC screening test at the correct interval was associated with a higher prevalence of current screening in that region (OR, 1.19; 95% CI, 1.05-1.37).

Seventy-one percent of physicians who correctly indicated that screening should begin at age 50 reported recommending at least one test at the correct interval. Of these physicians, 54.4% recommended initiating screening at age 50. Overall, 37.4% of physicians correctly recommended both initiation and frequency.

## Discussion

Our analysis adds to earlier work demonstrating regional variation in the use of CRC screening (13) by examining the relationship between CRC screening use and the recommendations of primary care physicians, by hospital-referral region. Although higher proportions of physicians who correctly recommend CRC screening were associated with relatively small changes in the proportion of adults screened, increases in correct recommendations would result in many more people being screened. For example, if in each hospital-referral region the proportion of primary care physicians who recommend initiating screening at age 50 years increased by 30 percentage points, an estimated 1.5 million additional adults older than 50 years would be screened (based on an estimated U.S. population of 77 million older than 50 years, derived from the U.S. Census [www.census.gov/popest/national/asrh/NC-EST2005/NC-EST2005-01.xls]). Similarly, a 30-percentage-point increase in the proportion of primary care physicians in each region who recommend screening at the correct interval could result in an additional 2.1 million people being current on screening.

Our work is consistent with earlier work suggesting that lack of provider counseling about CRC screening, rather than poor patient acceptance, is associated with lower rates of screening (28,29). Patient recall of physician's recommendations is one of the strongest predictors of cancer screening (8-11,30). Our findings suggest that population-based interventions directed at the CRC screening recommendations of primary care physicians may improve CRC screening use. Despite the endorsement of several influential national organizations and an awareness of the importance of CRC screening, however, many primary care physicians report screening practices that are inconsistent with the guidelines (12). Practice guidelines alone may be limited in their effect on physician behavior for several reasons, including lack of awareness, lack of agreement with the recommendations, barriers to successfully implementing the guideline, and concerns about patient acceptance of the guideline (31).

Several studies suggest that office-based systems may improve the prevalence of CRC screening in primary care practices (4,32-34). One successful example, which was intended to increase cancer screening among disadvantaged patients, was based on the assignment of office responsibilities and the use of a cancer-screening checklist with chart stickers (35). An intervention requiring quarterly feedback of a provider's CRC screening rates was also associated with increases in screening (36,37). Although some studies suggest that local, practice-based physician-reminder systems may improve the delivery of CRC screening and other types of cancer prevention (4,32,33), our results suggest a role for regional interventions to increase provider compliance with guidelines. Information on the feasibility of these types of interventions is limited, however, and one quality improvement program implemented by a managed care health plan to increase CRC screening was not successful (38). Outreach and education by leaders in medical opinion (i.e., academic detailing), however, have been shown to improve adherence to guidelines for preventing myocardial infarction and other medical conditions (39).

Our analysis has several limitations. The data do not allow us to examine the relationship between the recommendations of an individual's personal physician and that individual's screening behavior, and they are not intended to be a proxy for the recommendations of a specific physician. Rather, our findings reflect regional differences in physician recommendations. Although both the NHIS and the SCCSP are nationally representative, we included only respondents who lived in hospital-referral regions that were sampled in both surveys, and our results may not be generalizable to individuals in other areas. Finally, although we selected data from the NHIS that were collected several years after the SCCSP data that we used, some of the individuals in the NHIS may have been screened before the SCCSP was conducted. Unfortunately, NHIS does not allow identification of the precise year of a test.

Our findings indicate that regional differences in the recommendations of primary care physicians for CRC screening are associated with differences in screening use by individuals. For this reason, increasing the use of CRC screening in the United States may require interventions to improve the beliefs and recommendations of primary care physicians about CRC screening.

## Figures and Tables

**Table 1:** Factors Associated With Ever Having Been Screened for Colorectal Cancer (CRC), United States^a^

Factor	N	Ever Screened n (Weighted %)	Adjusted OR^b^ (95% CI)
Total	12,727	6289 (50.2)	NA
**Sex**			
Male	5280	2582 (50.9)	Ref
Female	7447	3716 (49.6)	0.95 (0.86-1.05)
**Race/ethnicity**			
Non-Hispanic white	8362	4598 (54.6)	Ref
Non-Hispanic black	1981	898 (46.9)	0.94 (0.82-1.08)
Hispanic, other race/ethnicity	2384	802 (33.6)	0.66 (0.56-0.78)
**Education^b^**			
<High school graduate	3054	1114 (36.5)	0.52 (0.43-0.62)
High school graduate	3576	1718 (47.7)	0.69 (0.59-0.79)
Some college	3029	1696 (56.6)	0.93 (0.80-1.09)
College graduate	2914	1721 (59.1)	Ref
**Health insurance^b^**			
Uninsured	1036	227 (22.0)	0.54 (0.43-0.69)
Medicare with private supplemental insurance, or private insurance	8495	4658 (54.9)	Ref
Medicare without supplemental insurance	1881	921 (50.1)	0.81 (0.69-0.94)
Medicaid or dually eligible for Medicare with Medicaid	1260	479 (37.0)	0.57 (0.47-0.68)
**Usual source of care^b^**			
Yes	11,775	6120 (52.6)	Ref
No	754	115 (15.0)	0.31 (0.24-0.41)
**Ever diagnosed with cancer other than CRC^b^**			
Yes	1463	959 (64.6)	1.38 (1.20-1.58)
No	11,251	5335 (48.3)	Ref
**Number of chronic diseases**			
0	3930	1480 (39.2)	1.35 (1.28-1.42)
1	3994	2001 (51.1)	
2	2791	1571 (57.6)	
≥3	2012	1243 (62.4)	
**Behavioral risk factors^b^**			
0	2663	1593 (60.3)	0.78 (0.72-0.85)
1	7627	3675 (48.8)	
≥2	2110	914 (43.3)	
**Primary care physicians in HRRs recommending CRC screening beginning at age 50 y**			
0%-29%	1157	533 (47.3)	1.09 (1.01-1.18)^c^
30%-49%	3966	1947 (50.0)	
50%-59%	3636	1767 (50.3)	
60%-69%	1890	929 (49.2)	
≥70%	2078	1122 (54.8)	
**NHIS participation year**			
2000	6238	2982 (48.0)	Ref
2003	6489	3316 (52.0)	1.15 (1.05-1.26)

OR indicates odds ratio; CI, confidence interval; Ref, reference group; NA, not applicable; HHR, hospital-referral region.

a Analysis of data associated with hospital-referral regions (HRR) from the National Health Interview Survey (2000, 2003) and the Survey of Colorectal Cancer Screening Practices, Primary Care Physician Questionnaire (1999–2000).

b Data missing for education (n = 154), health insurance (n = 55), prior diagnosis of cancer other than CRC (n = 13), usual source of care (n =198), and behavioral risk factors (n = 327). Models adjusted for age, sex, race and ethnicity, education, insurance, usual source of care, prior diagnosis of cancer other than CRC, number of chronic health conditions, number of behavioral risk factors for colorectal cancer, year of NHIS survey participation, and proportion of primary care physicians in HRRs who recommend CRC screening beginning at age 50 years.

c Odds ratio expressed for a 30-percentage-point increase in the proportion of primary care physicians in an HRR who recommend CRC screening beginning at age 50 years.

**Table 2:** Factors Associated With Being Current on Colorectal Cancer (CRC) Screening, United States^a^

Factor	N	Current Screening n (Weighted %)	Adjusted OR^b^ (95% CI)
Total	6298	4893 (77.9)	NA
**Sex**			
Male	2582	2063 (80.3)	Ref
Female	3716	2830 (75.8)	0.76 (0.65-0.90)
**Race/ethnicity**			
Non-Hispanic white	4598	3570 (78.5)	Ref
Non-Hispanic black	898	688 (74.8)	0.99 (0.76-1.28)
Hispanic, other race/ethnicity	802	635 (77.1)	1.09 (0.80-1.49)
**Education^b^**			
<High school graduate	1114	846 (74.1)	0.80 (0.60-1.05)
High school graduate	1718	1322 (77.4)	0.88 (0.71-1.10)
Some college	1696	1282 (76.4)	0.78 (0.62-0.99)
College graduate	1721	1407 (81.8)	Ref
**Health Insurance^b^**			
Uninsured	227	154 (66.7)	0.82 (0.51-1.32)
Medicare with private supplemental insurance	4658	3656 (78.5)	Ref
Medicare without supplemental insurance	921	707 (77.5)	1.04 (0.81-1.32)
Medicaid or dually eligible for Medicare with Medicaid	479	369 (77.9)	1.17 (0.81-1.69)
**Usual source of care^b^**			
Yes	6120	4786 (78.4)	Ref
No	115	61 (47.1)	0.28 (0.17-0.47)
**Ever diagnosed with cancer other than CRC^b^**			
Yes	959	792 (82.1)	1.26 (0.98-1.61)
No	5335	4098 (77.2)	Ref
**Number of chronic diseases**			
0	1480	1122 (76.0)	1.02 (0.95-1.10)
1	2001	1563 (79.5)	
2	1574	1234 (78.1)	
≥3	1243	974 (77.4)	
**Behavioral risk factors^b^**			
0	1592	1276 (80.4)	0.93 (0.82-1.05)
1	3675	2854 (78.1)	
≥2	914	685 (73.9)	
**Proportion of primary care physicians in HRRs recommending at least one CRC screening test at the recommended interval**			
0%-29%	86	62 (70.7)	1.19 (1.05-1.37)^c^
30%-49%	495	376 (76.7)	
50%-59%	1546	1176 (76.3)	
60%-69%	1468	1136 (77.6)	
≥70%	2703	2143 (79.9)	
**NHIS participation year**			
2000	2982	2242 (74.1)	Ref
2003	3316	2651 (80.8)	1.45 (1.22–1.73)

OR indicates odds ratio; CI, confidence interval; Ref, reference group; NA, not applicable; HRR, hospital-referral region.

a Analysis of data associated with hospital-referral regions (HRR) from the National Health Interview Survey (2000 and 2003) and the Survey of Colorectal Cancer Screening Practices, Primary Care Physician Questionnaire (1999–2000).

b Data missing for education (n = 49), insurance (n = 13), usual source of care (n = 63), prior diagnosis of cancer other than CRC (n = 4), and behavioral risk factors (n = 117). Models adjusted for age, sex, race and ethnicity, education, insurance, usual source of care, prior diagnosis of cancer other than CRC, number of chronic health conditions, number of behavioral risk factors for CRC, year of NHIS survey participation, and proportion of primary care physicians in HRRs who advised at least one CRC screening test at the recommended interval.

c Odds ratio expressed for a 30-percentage-point increase in the proportion of primary care physicians in an HRR who recommend at least one CRC screening test at the recommended interval.
